# Autonomy conquers all: a thematic analysis of nurses’ professional judgement encountering resistance to care from home-dwelling persons with dementia

**DOI:** 10.1186/s12913-022-08123-x

**Published:** 2022-06-04

**Authors:** Åshild Gjellestad, Trine Oksholm, Herdis Alvsvåg, Frøydis Bruvik

**Affiliations:** 1 Faculty of Health Studies, VID Specialized University, Bergen, Norway; 2 Department of Global Public Health and Primary Care, University of Bergen, Bergen, Norway

**Keywords:** Resistance to care, Forced treatment and care, Involuntary care, Person-oriented care, Home care, Home health care, Decision-making, Qualitative, Interview

## Abstract

**Background:**

Adequate care support from home health care nurses is needed to meet the needs of an increasing number of home-dwelling persons with dementia and those who resist care. The decisions nurses make in home health care when encountering resistance from persons with dementia have an extensive impact on the quality of care and access to care. There is little research on what influences nurse’s encounters with resistance to care from home-dwelling persons with dementia.

**Research aim:**

To get insight into how nurses experience resistance to care from home-dwelling persons with dementia.

**Methods:**

A qualitative research design using a thematic analysis was conducted following the six steps by Braun and Clarke. Data was gathered from three focus group and three individual interviews, and a total of 18 nurses from home health care participated. The interviews took place over a period of 5 months, from December 2020 to April 2021.

**Ethical considerations:**

Approved by the Norwegian Centre for Research, reference number 515138 and by the research advisers and home care managers in each section of the municipality.

**Results:**

Two main themes were identified: 1) Challenged by complex and inadequate care structures and 2) Adapting care according to circumstances. There were three subthemes within the first main theme: lack of systematic collaboration and understanding, insufficient flexibility to care, and the challenge of privacy. In the second main theme, there were three subthemes: avoid forced treatment and care to protect autonomy, gray-areas of coercive care and reduced care. The two main themes seemed to be interdependent, as challenges and changes in organizational structures influenced how nurses could conduct their care practices.

**Conclusion:**

Our findings indicate that nurses’ responsibility to decide how to conduct care is downplayed when facing resistance. Further, their professional judgement is influenced by contextual factors and characterized by a strong commitment to avoid forced treatment and care.

A continuous challenge is to safeguard shared decision-making at the same time as it is balanced against risks of severe health damage in home-dwelling persons with dementia. A fundamental question to ask is whether autonomy does conquer all, even when severe health damage is at stake.

## Introduction

Dementia care is primarily undertaken in the community, and adequate care support from home health care nurses is often needed to meet the care needs of the increasing number of home-dwelling persons with dementia [1–4]. Dementia affects the person’s ability to take care of themselves [5]; it affects cognitive functions and is often followed by behavioral and psychological symptoms, such as aggression, agitation, anxiety, irritability, depression, apathy, delusions, hallucinations, disinhibition, or lability, motor disturbance and night time behaviors [5, 6]. These symptoms are often understood as aggressive behavior. This form of communication from the person with dementia is associated with resistance to care and increased use of restraint [7, 8]. Resistance to care, understood as verbal objection or physical objection or escape, often increases when dementia progresses [9]. Increased need for assistance to care for persons with dementia has been associated with the use of restraint or involuntary treatment. Previous research has demonstrated that experiences with forced treatment and care are common in home health care [8–11]. How nurses respond when encountering resistance has an extensive impact on persons with dementia’s quality of care and access to care [10–12]. Home health care services have experienced a transfer of responsibilities from hospital care to community care, but with limited resources [13]. The ethical challenges of the allocation of services and priority settings are well known [13]. What complicates home health care is that it becomes intertwined with the person’s home social life [14]. There is little research on nurses’ clinical decision-making in situations where home-dwelling persons with dementia resist care [12, 15]. Moreover, nurses’ responsibilities in prioritization are seldom emphasized in overarching policy documents [16]. When encountering resistance to care from persons with dementia, good decision-making and transparency of these decisions are important.

## Background

Factors like capability, opportunity, and motivation may influence nurse’s behavior in the physical, social, and political context [17–19]. Behavior has also been found to be influenced by traditional professional practices and hierarchical structures [20]. When encountering resistance to care, nurses’ behavior and response to the situation may therefore be influenced by many factors, including attitudes toward care, knowledge of care, reasons for restraint, and ethical principles [8, 21]. The nurses’ experience, knowledge of the patient, the relationships involved in the care process, and the context of the situation are other influencing factors [21, 22].

Procedural law expressed in workplace policies and governmental policy documents have been found to influence nurses’ decision-making practices [19]. Health professionals are expected to behave according to current policies and ethical standards, and it is a strong underlying assumption that patients should be active and involved in their own health and health care decisions [16, 23]. Patient autonomy can be defined as letting the patient make well-informed and free choices based on their own values [13]. However, the concept and assessment of autonomy in persons with dementia is complicated and needs to be balanced against dignity and vulnerability in dementia-care. When cognitive impairment due to dementia progresses, it may be difficult to balance a person with dementia’s right to self-determination and the duty of professional care responsibilities [24]. Development of self-neglect due to progression of dementia may lead to lack of recognition of need for personal hygiene [25]. Legislation exist to guide health professionals’ when encountering resistance to care from patients who cannot make informed choices due to lack of capacity to consent [26].

From previous research we know that the reported use of forced treatment to care for persons with dementia in home health care is low and there is little knowledge about how health professionals face situations of resistance [3]. The prevalence of forced treatment and care found in international studies show considerable variation [12, 27]. Previous research has also demonstrated that balancing safe care with the person’s integrity is a main concern when applying trust-building interventions to home-dwelling persons with dementia that resist care [28]. Further, a recent systematic review of ethical challenges in home health care argues that there is a need for research on nurses’ priority-setting decisions in daily care [13]. The rationale for conducting this study was to develop knowledge and to get insight into how nurses working with persons with dementia in home health care encounter resistance to care and how they make care decisions in these challenging situations.

### Research aim

To get insight into how nurses experience resistance to care from home-dwelling persons with dementia

## Study design

A qualitative research design using focus group interviews and individual interviews was chosen for this study. Inspired by critical realism we used a thematic analysis approach to analyze the collected empirical material. When exploring human experiences and when interpretating written text from interviews a qualitative design is considered a suitable approach [29].

### Setting

Home health care requires the collaboration of health professionals, patient and family. In Norway each patient has been assigned a general practitioner who is required to work together and collaborate with nurses in the municipal home health care team. The municipality carries the main responsibility in facilitating collaboration. However, the general practitioner is also, by law, expected to collaborate and follow up their patients. Together the nurses in home health care and the general practitioner form the main part of the patients primary health care services [30].

### Sampling

A purposive sample was used in this study. We were interested in the experiences of nurses working closely with home-dwelling persons with dementia, and we therefore included participants that were either licensed practice nurses or registered nurses and that had present or recent experience in giving care to home-dwelling persons with dementia. In Norway, licensed practice nurses have their education at the high school level with a specialization in health and social care. Registered nurses are educated at the bachelor level. The nurses participating in the study worked in community home health care with allocated lists of patients where tasks were based on the person with dementia’s health care needs and were predefined by the sectoral case management office of the municipality. The participants are hereafter referred to as nurses.

### Sample size

A total of 18 nurses, divided into three focus-groups interviews and three individual interviews from home health care participated. The decision of sample size was based on methods literature describing sample size where a rule of thumb is to plan for three - four focus groups. We completed three focus-group interviews and three individual interviews [30]. .

### Method of approach

The participants were recruited from municipal home health care services in different geographical zones within one municipality. The first author contacted the municipality and was provided with a research coordinator who contacted special nursing advisers of the relevant home health care departments. Possible participants were identified and contacted by the special nurse advisor in the particular department. Interested health professionals received a personal invitation letter with information about the study and an informed consent form to sign before the focus group interview. In collaboration with the contact persons, an appropriate time and location for the focus-group interviews were chosen. For the individual interviews, the first author agreed with the participant directly. The consent forms were returned directly to the first author in paper or electronically as a scanned document.

### Data collection

The focus-group interviews took place at reserved rooms located at the participants workplace. There were four to six participants in each focus-group interview. The three individual interviews were conducted by telephone

The first author (ÅG) was the first moderator (M1), and the three supervisors assisted as co-moderator (M2) in one focus-group interview each. The individual interviews were all conducted by the first author (ÅG)

The topics introduced in both the focus groups and individual interviews were formed as open-ended questions and allowed the participants to discuss the factors they considered relevant

**Fig. 1:**
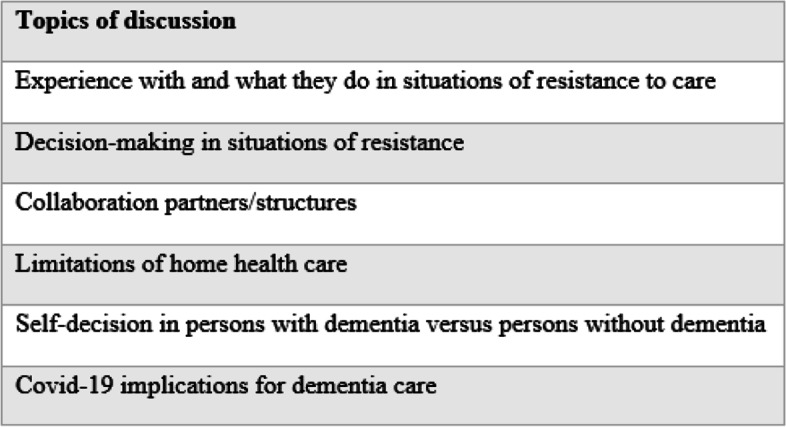
Main topics of the interview guide

A semi-structured interview guide was developed with a reference group that consisted of health-professionals working in home health or/and within dementia care, researchers of elderly care, and representatives from dementia organizations, and a research team with extensive experience in qualitative studies and dementia care. The interview guide was pilot tested with three registered nurses from the reference group to check for relevance and understanding of the questions and to increase validity of the findings. An overview of the main topics introduced is presented in Fig. 1.

The practical implementation of the interviews was delayed due to restrictions of the COVID-19 pandemic and all three focus-group interviews were postponed from their original date. The intended fourth focus-group was exchanged with three individual telephone interviews due to fear of contagion if mixing staff. The interviews took place over a period of 5 months from December 2020 to April 2021.

In the focus-groups we used two tape recorders to assure the audibility of recordings, and we used name tags to enhance communication within the group. The individual telephone interviews were adapted to the preferred time of the participant and were recorded using a digital voice recorder. Each interview (focus and individual) lasted between 70 and 90 minutes.

### Analysis

The interviews were transcribed verbatim by the first author (ÅG) and an external transcriber. The external transcriber signed an agreement of confidentiality and non-disclosure. ÅG transcribed two focus group interviews, and the external transcriber transcribed one focus-group interview and the three individual interviews. A drawing of where the participants were seated was made to facilitate transcription and separation of voices of the focus groups. All coauthors read the transcriptions and were part of the analysis process.

**Fig. 2:**
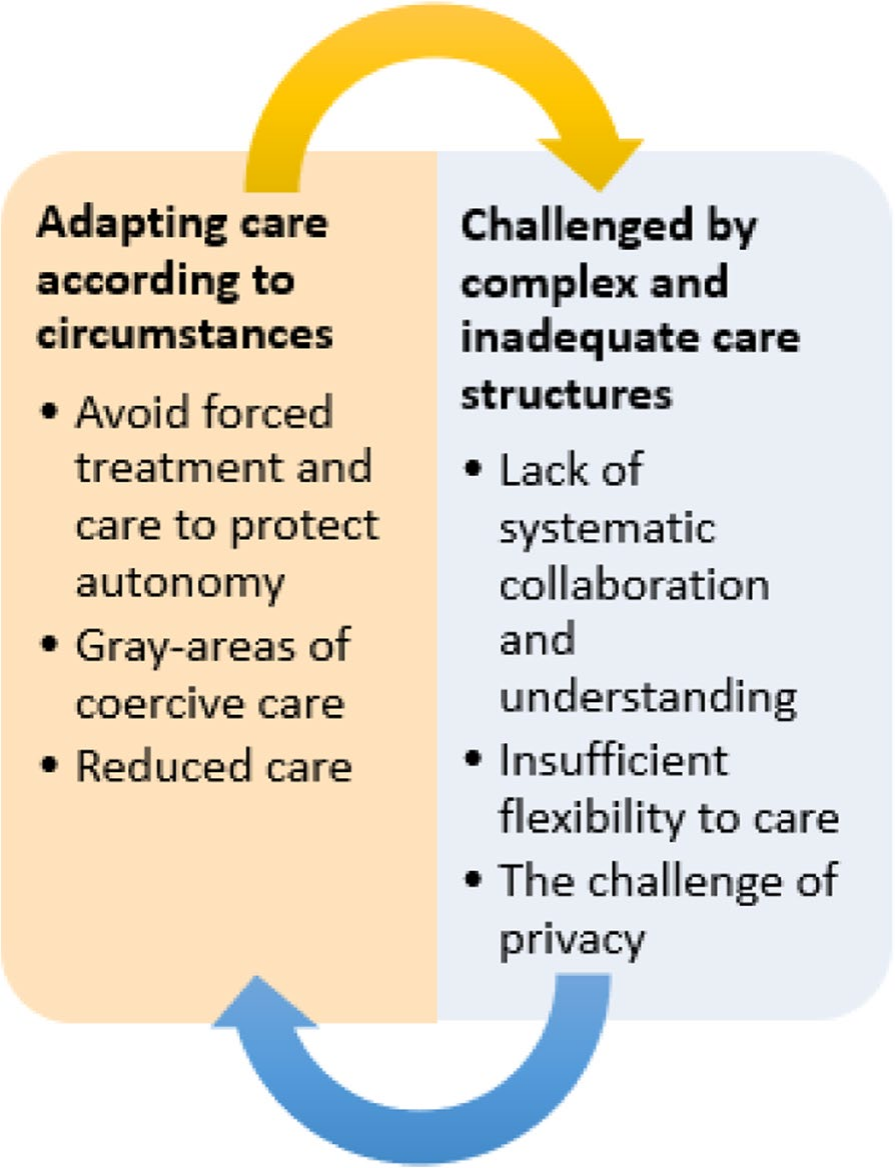
Final themes of nurses’ experiences when encountering resistance to care

Inspired by critical realism, a qualitative inductive thematic analysis was conducted following the six steps by Braun and Clarke: step 1) become familiar with the data, step 2) generate initial codes, step 3) search for themes, step 4) review themes, step 5) define themes, and step 6) write-up [31]. The following explains more on the above six steps: 1) To obtain a general impression and to become familiar with the material, the first author, (ÅG) read through all the transcribed interviews several times, searching for patterns and meanings. Transcripts were confirmed with the original audio recordings for accuracy. The coauthors (TO, HA, FB) individually read all the interviews, with a particular emphasis on the interview they participated in to search for patterns and meanings and initial ideas for coding. We evaluated that saturation was met after completion. 2) The research group met to discuss meaning, patterns and coding, and an initial discussion of possible themes. Notes were written in the margin, and colors were used to mark patterns for coding. 3) The list of codes was analyzed by the first author (ÅG) and sorted into groups that belonged together. Potential themes, main themes and subthemes were identified. 4) The themes were reviewed with the coauthors in a back-and-forth process between themesand the dataset continued until the names of the themes expressed the content and meaning of the data in a suitable and relevant way. The analysis was an iterative process, and we moved from the context of the text to the individual parts of the text, allowing the parts to inform each other. The first author double checked transcriptions against recordings when using citations. 5) Final themes were utterly defined, refined, and named. 6) The final themes were written out in a manuscript. During this process, the themes were reviewed by two research groups. The first author, (ÅG) received important feedback, and a new round of refining was conducted. An overview of the final themes is provided in Fig. 2.

### Translation procedures

The analysis of the empirical material was conducted in Norwegian. When themes and subthemes were decided the first author in collaboration with the research team translated them into English. The quotes that we decided to use in the manuscript were subsequently translated into English. During translation, text quotes were translated verbatim and then modified to obtain equivalence in meanings and interpretations. All authors read both the Norwegian and the English quotes. The quotes used in the manuscript were marked to identify which nurse and focus group it came from to make it possible to go back to the data and double-check for relevance.

### Ethical considerations

Approval to conduct the study was given from Norwegian Centre for Research Data with reference number 515138/2020. The study handles sensitive topics for the health professionals involved. During the process of analysis, the first author was the only one who knew the identity of all participants. Therefore, the names associated with the nurses’ quotes were anonymized to protect the identities of the nurses. The findings are presented in such a way that neither the participating home care staff nor the persons with dementia they assisted can be identified.

## Results

The participants in this study were all female, had an average of 19 years of experience, and 13 of them had continued education relevant to care for persons with dementia (Table 1)

**Table 1:** Participants background in focus group and individual interviews

Participation	Age mean (range)	No. of participants	Years of experience in HHC mean (range)	Education	Participants continued education^a^
Focus group 1	55.5 (46–62)	6	27 (20–35)	Licenced practice nurses	6
Focus group 2	52.4 (44–61)	5	18.6 (7–32)	3 registered nurses 2 licenced practice nurses	1 2
Focus group 3	49 (43–57)	4	9.5 (3–18)	Registered nurses	1
Individual interviews	50 (34–66)	3	17 (2–31)	Registered nurses	3
Total	52 (34–66)	18	19 (2–35)	10 registered nurses 8 licenced practice nurses	13

^a^Continued education included: ABC dementia course (4), geriatrics (3), psychiatry (1), palliation (1), nutrition (1), pedagogics (1), intensive care nurse (1), master of evidence-based practice (1)

When we analyzed the empirical data two main themes were identified: 1) challenged by complex and inadequate care service structures, and 2) adapting care according to circumstances. There were three subthemes within the first main theme: lack of systematic collaboration and understanding, insufficient flexibility to care, and the challenge of privacy. In the second main theme, there were also three subthemes: avoid forced treatment and care to protect autonomy, gray-areas of coercive care and reduced care. The two main themes seemed to be interdependent, as challenges and changes in organizational structures influenced how nurses could conduct their care practices. Equally, the choices made by individual nurses when adapting care to the circumstances of the person with dementia would have consequences for the resources available to other patients and the structural work environment of colleagues (Fig. 2).

### Challenged by complex and inadequate care structures

The nurses described that they were challenged by complex and sometimes inadequate care structures. They lacked systematic collaboration and mutual understanding with the general practitioners and nurse leaders in situations of resistance to care. The lack of communication and support when facing resistance influenced their ability to provide good dementia care.

#### Lack of systematic collaboration and mutual understanding

The nurses reported that there was a lack of systematic collaboration within the care system. They especially reported lack of feedback from management. In one focus group, they described to have reported notices of deviations related to home care visits for years without receiving any feedback from the management. There were also challenges with collaboration with the patient’s assigned general practitioner.

The nurses described the collaboration with the general practitioners as crucial. The nurses mainly communicated with the practitioners using digital e-message communication. The response time varied from 1 h to non-existent. The collaboration was ad hoc, from patient to patient, and situation to situation. Some general practitioners did home visits to examine the situation; however, this was not a common practice. The nurses found themselves in extremely demanding situations where they continued to provide help for long periods of time.

*She has not showered for over a year, and her self-care is poor. This is a woman who previously has been very preoccupied with appearance. And even when she lets you assist with hand wash, you may use 45 minutes just to make her do it. She manages to do it on her own, but we know it does not happen.* (Individual interview-2)

There was a common understanding that only the patients’ general practitioner could make decisions regarding forced treatment and care, but they would seldom make such decisions. Overall, the lack of collaboration with general practitioners was challenging.

*Nurse 1: It is a desperate situation. We have several general practitioners that are difficult to cooperate with. Either we do not get an answer, or they do not answer what we ask them. We really do not know what to do about it. Nurse 4: Every district should have a chief general practitioner of home health care services.* (Focus group-3)

This lack of collaboration led to stalled situations where nobody would make a needed decision. We found that the nurses called for a more systematic collaboration around decisions for these patients before the situations became severe. They described a need for a structure that enabled the development of common knowledge and strategies for dementia care, such as challenging situations of resistance. The inadequate structures implied that challenging care situations continued with risks of undocumented coercion or neglect.

The nurses experienced that the general practitioners often do not share the understanding of the gravity of the patient situations. The nurses emphasized that they were in the position to observe the patients in their daily routines, however the nurse’s autonomous responsibility and accountability to decide how to conduct care seemed to be downplayed.

*Nurse 4: It is the general practitioner who is responsible for them, but they do not see the patients when they need help with personal hygiene or in other similar situations.* (Focus-group-1)

#### Insufficient flexibility to Care for Persons with dementia

The nurses described that having a “time account” was crucial when persons with dementia resist care. A time account enables the possibility to use continuous visits over time to warm up the relationship with the person with dementia and to be flexible with time during the visits. The average time needed to help was approximately 45 minutes, depending on the situation. However, for many of the nurses, this was not always possible because of the high number of patients on their list each day.

*Nurse 2: If I have a list of 15 patients and, if six of them have dementia and resist help with ADL in the morning, then...Nurse 1: you do not have time … Nurse 2: We can’t make it! It’s a fact, we do not have enough time to spend with the patients with dementia after they were included in the group of patients where persons with dementia are mixed with patients that are cognitively clear.* (Focus-group 3)

The nurses stated that limited time and lack of staff were common challenges faced in home care. Whenever persons with dementia resist care, some nurses contemplated whether to try again the same day, but it was dependent on the ability to reorganize or decrease the workload. Their experience was that going back did not concord with the time limit within their lists. Moreover, they did not have a culture or routines that encouraged them to do so. On the positive side, many nurses reported collaborating with the next shift nurse or trying to complete the task the next time they came to work.

All the nurses reported working within a “primary nursing” care system, where a named nurse assumed responsibility for a patient’s plan of care. The intention of having this system was that the nurse could holistically plan for care, collaborate with family, and be flexible to grant patient wishes and needs. However, the nurses reported that the system had limitations. Some nurses described that they seldom had the chance to follow up on their patients regularly because of lists and needs shifts. It could be weeks between each visit. On the other hand, others described it to be rather demanding to go to the same patient often. However, these descriptions were a little different for nurses who worked within the dementia team. They reported having a greater flexibility opportunity.

During emergencies, the nurses would call either the practitioner or the emergency room. The possibility of admission to a short stay at a nursing home was also an important tool when the situation became severe. The nurses described that these transitions were rarely carried out or documented as coercion, even if the person with dementia resisted.

COVID-19 only made existing challenges of the care structure more visible. Structures for collaboration worsened due to meeting restrictions. The possibility of fellow reflection and problem-solving became limited. All nurses reported that systematic structures for collaboration had diminished during Covid-19.

#### The challenge of privacy

Nurses in home health care emphasized the advantages of caring for persons with dementia at home, such as increased quality of life, feeling safe, and familiar surroundings. The nurses experienced positive collaboration with other members of society like police officers, taxi drivers, grocery store workers and family members. However, there were also some unique challenges in private home care that could be difficult for the nurses to endure.

One of the difficulties working in the patients’ home was that nurses perceived that they violated the person with dementia’s right to privacy and to decide in their own homes. They described the difficulty in caring for patients in filthy and undignified living surroundings. The feeling of invading privacy was strengthened due to the experiences of being verbally and physically attacked. The nurses all considered it more damaging to give forced care in a private home than in a nursing home.

Being an observer of these situations could be emotionally draining. Nevertheless, they seemed to have an understanding that for many persons with dementia, it was best to remain living at home.

*To him, it is quality of life in living at home. Even if we, in our standards, do not feel the same way. We need to see it from the patient’s perspective.* (Focus-group 2)

Another challenging factor of going into a private home was the need to relate to the family members. On one hand, the nurses emphasized that family members were their most important collaborators, but at the same time, family members could have high expectations of what the nurses should accomplish. They could also have differing understandings of what kind of care was needed.

*Nurse 1: I remember it as challenging that the husband was there, complaining to us that she needed to be taken to the toilet. He told us about episodes where she had been soaking wet. I remember it as a difficult situation because it was so degrading to her.* (Focus-group 3)

### Adapting care according to circumstances

We found that avoiding coercion and providing adequate care through building trust were the main care principles when encountering resistance from persons with dementia. However, the results also demonstrated that consequences of resistance to care also embedded gray areas of coercive care, and reduced care.

#### Avoid forced treatment and care to protect autonomy

It was a general understanding among the nurses that they should and could not use coercion. The avoidance of coercion was important and strongly embedded in the cultural as well as formal guidelines for acceptable care behavior within home health care. The nurses were aware that formal decisions of forced treatment or care could be made, but this option was seldom used, and they did not seem to have an understanding that they could be responsible for such a decision.

When encountering resistance, the nurses considered the possible damages due to forced care to be higher than the risks of missed health care. If they were stopped by the person with dementia at the door, they assessed the risk of not being let back in if they used any kind of force. The nurses reported that they managed to solve such difficult situations of care without using force to avoid use of involuntary interventions.

*We need to use time and make them feel safe, hold their hands, and let them say stop when they feel it is too much. You need to work, work, and work. It requires a lot of time, and it is occasionally successful. If not today, then another day. But we had one patient who always said no. In that situation, we tried to get a decision of forced treatment and care.* (Focus group-1)

#### Gray-areas of coercive care

However, nurses also described that there were gray areas. Several nurses stated that they sometimes did cross the line and used forced treatment and care even if a decision of this had not been made. In one focus group, a particular situation came up where the nurses emphasized that it would have been wrong not to intervene.

*Nurse 3: Of course, we have to respect it when they do not want help. But when we come to a patient that has had feces and they do not recognize their own good, then we do it by force anyway, because it would be wrong not to do it. Are they supposed to just lie there? We have crossed the lines occasionally, but they are grateful afterwards.* (Focus group-1)

When the nurses discussed what they did when they encountered resistance, they mentioned interventions such as the use of sedative medication. This was used to reduce resistance prior to transferring a patient from the chair to the bed. Signatures from persons with dementia were used to consent to use door alarms or to make deals with the patient to accept a nursing home admission, even if the patient would later resist staying inside or moving to the nursing home. These interventions were seldom documented as forced treatment and care but were found to be needed with the aim of caring for person with dementia.

#### Reduced care

We found that when persons with dementia resisted care, the nurse’s main approach was pragmatic, attempting to provide adequate care, although not always successful. It seemed that a common consequence of resistance was reduced care.

*Nurse 1: We do not stand outside their door and say: I am here to help you with the shower. That is something we would never do. We go inside, we sit down, we talk, and then we get to the shower in time. Nurse 2: I have a female patient, now we are allowed to assist her with ADL, but before that, she did not shower for months. But we cannot carry them into the shower. In some situations, it just has to be as good as it gets. (*Focus-group 2)

They attempted to provide good care as much as possible considering the circumstances. One nurse elaborated on what was considered adequate care for one of her patients:

*Nurse 2: What is good enough? I have a woman that has been anorectic all her life. You have to tell yourself that this has to be as good as it gets. And if she keeps her weight, we have to be satisfied with that. You have to look at their whole life course and consider what you can accomplish. You cannot change that kind of attitude* (Focus-group 2).

The nurses reflected upon why the person resisted. One nurse described that resisting help could be a defense mechanism when the person with dementia experienced a loss of control but struggled to understand or to accept it. However, the nurses did not seem to systematically assess whether the person with dementia had the capacity to understand the need for health care when resisting to it.

## Discussion

The present study explored how nurses in home health care experience and encounter resistance to care from home-dwelling persons with dementia. Our findings demonstrated that the nurses were quite unified in their approach to care. Their main goals were to avoid coercion and to provide adapted care to persons with dementia who resisted care. However, we found that gray areas of coercive care and reduced care could also be a consequence of resistance. The nurses reported that care situations could become stalled when resistance to care was perceived as strong. The findings also indicated a lack of meeting points for professional discussions and support in challenging situations of resistance to care. Moreover, we found weak structures for collaboration and unclear division of responsibilities between general practitioners and nurses when home-dwelling persons with dementia resisted help. The nurses did not describe or perceive themselves as the responsible decision-makers of nursing care in challenging situations and their autonomous responsibility to conduct decisions of care was downplayed. Surprisingly, the nurses reported not using forced treatment and care when encountering resistance.

### The need for structures that enable multidisciplinary collaboration and clarify responsibility

In the present study, the nurses called for structures that enabled collaboration with the general practitioners. The nurses’ strong emphasis on collaboration in the present study is in line with impositions of multidisciplinary decision-making in legal and clinical guidelines to safeguard the best interest of the patient in difficult situations of care [32, 33]. Previous studies have reported that staff in home care experience dilemmas when left alone to make difficult judgments of when and how to act in risky situations [34]. Furthermore, a previous study found that a lack of recognition of nurses’ roles and their nursing expertise by other health care providers hampered their decision-making ability [35]. Moreover, nurses seek the opinions of other professionals, patients, and family, and are influenced by context when making decisions [24, 32]. The importance of nurses’ collaboration with other providers of health care, the pressure and lack- of-recourse- situation and its implications for patient safety has been well documented in previous research, and joint leadership in primary care has been argued for [36, 37]. Nurses serve as a bridge between patients, family members and other health professionals and need to maintain communication with all parties and provide quality care [33]*.* However, the increased emphasis on collaboration also may have had unforeseen consequences of weakening accountability and that the room available to act in autonomous decisions of nurses has become smaller. The nurses also called for structures that enabled communication with other nurses. They had few meeting points, and when encountering ethically or clinically challenging situations, they consulted other nurses mostly through phone calls, during lunch or before starting work. Moreover, communication had decreased in recent years due to the removal of oral reports and as a result of meeting restrictions during COVID-19. Previous research has demonstrated a need for support when facing ethically challenging situations of care [35, 38]. This has been politically acknowledged. Where clinical ethics committees have been established in municipalities, challenges of coercion has been among the most common topics discussed [39]. Our study supports previous research that has outlined complex ethical dilemmas combined with a scarcity of recourses in home health care and the difficult decisions that have to be made there. However, if nurses are to fulfill their intended role, they need to have organizational structures that support them, including communication with the general practitioners and peers in challenging situations of resistance to care.

Another important finding was the perceived division of responsibility. Nurses in our study did not describe or perceive themselves as the responsible decision-makers of nursing care when facing resistance to care, although they considered themselves the responsible care takers of the person with dementia. Previous studies report that nurses are increasingly in charge of complex care provided in home health care, i.e., regarding personal hygiene, nutrition, prevention of falls, and patient needs for supervision [34, 40]. In challenging situations of resistance to care, the nurses in the present study reported that these decisions should be made by the patient’s general practitioner. Previous research has found diverging results with regards to who is involved in forced treatment and care. In a systematic review Scheepmans et al. (2018) found that a common factor in all the studies was the importance of the role of family or informal caregivers [27]. They often requested or initiated the use of restraint, and family was an important part of the decision-making process. Nurses were the second group of people that often initiated restraint use. General practitioners were less involved, their roles being unclear, and largely limited to the prescription of medication to control the patient’s behavior, although some home care nurses preferred them to take a more active role in the decision process [27]. In the present study, there was unclarity regarding legal guidelines for decision-making when met with resistance to care and how responsibility should be divided between nurses and the general practitioners. There is a general expectation in professional nursing that nurses decide how to conduct care. Additionally, Norwegian legislation states that nurses may be the professional responsible for coercive nursing care decisions when the person is unable to make health care decisions for themselves due to lack of capacity to consent [26]. Moermans et al. (2018) found that general practitioners (47%) more frequently requested the use of involuntary treatment, but that nurses (81%) mostly applied it [11]. The present study indicated that the nurses perceived responsibility did not extend to situations of resistance and assessment of involuntary care. Not only can this be a threat to nurses’ professional autonomy, but it can also threaten the wellbeing and safety of persons with dementia living at home.

Our findings demonstrate a need for increased emphasis on nurses’ responsibilities and prioritizations in overarching policy documents and in clinical practice, and this is supported by previous research [16, 41]. Agreement about responsibility is important to accountability, and unclarity of these matters may lead to no one taking final responsibility for challenging situations that can risk the development of unmet health needs and severe health damage in home-dwelling persons with dementia, which is not uncommon [4]. Such an agreement was absent in this study. One explanation for this could be that there is little emphasis, awareness and competence about coercion and legislation in home health care. This is supported by previous studies that found that nurses find the concept of restraint unclear and that there is confusion between restraints and safety measures [10]. Another explanation could be that it has been established within the organizational context that nurses have the power to care but not to decide. Research on behavior change illuminates this further, reporting three essential conditions that need to be present to enable behaviors, namely capability, opportunity, and motivation [17, 18]. Lack of competence hinders capability and power to act. Moreover, the findings indicated that the nurses also lacked structures for professional support, which could influence decisions in risky situations of care. Without supporting recourses like flexibility and true time, the nurses will have less opportunity to provide good dementia care. The lack of these previously mentioned conditions could be a barrier to nurses’ professional decision-making.

### Balancing autonomy and assessing risk

Surprisingly, and contrary to previous research, this study indicated that forced treatment and care was not frequently used by nurses in home health care [12, 27]. On the contrary, the findings reflect a strong cultural incentive of respecting the person with dementia’s wishes and avoiding forced care among nurses in home health care. The nurses in this study strongly dissociated themselves from using forced treatment and care. In line with previous research, the nurses reported that the emphasis of patient health-risks weighed against forced treatment and care as a topic was not common in discussions in the home health care setting [8]. A plausible explanation for the findings may be that the nurses obtained an optimal balance between the person with dementia’s preferences and the most suitable care to provide in such situations, considering the circumstances and degree of resistance. However, there may also be other explanations relevant to the findings that will be discussed below.

Respecting autonomy and assessing health-risk in persons with dementia often appears to be an unsolvable dilemma that the health care professional needs to endure and remain in. Assessing and acting on risks in persons with dementia is challenging and professionals, family and the person with dementia may understand risk differently [34, 42]. The value of autonomy is strong [43, 44], and nurses in the present study emphasized that they aimed to respect the person’s preferences, even when contrary to what most people would want for themselves. They assessed needs within the context of what was possible to achieve without using forced treatment and care, i.e., living under very unsanitary conditions. Previous research has argued that nurses who support persons with dementia who live at home need to increase their attention to how to assess, communicate, and manage risk [34, 42], and we add that they need to develop competence in how to balance these assessments with shared decision-making, self-determination, and the understanding of autonomy in persons with dementia. Based on the findings from our study, the nurses’ respect for the person’s preferences were enforced by working within their private homes. The findings illuminate that forced treatment and care within the privacy of a home was considered a threat to the nurse-patient relationship and was worse and more intrusive than using force in a nursing home. The consequence could be a ruined relationship where the health professionals would not be let back in. This is contrary to previous research that has found that nurses’ main preoccupation when applying forced treatment and care is to ensure the safety of persons with dementia [45].

Another interesting finding related to the understanding of autonomy in persons with dementia was that the nurses in this study did not give attention to assessment of capacity to consent. Assessment of capacity to consent is considered fundamental for patient rights when meeting resistance to care [26]. In the present study, the nurses reported that assessing capacity to consent was not something they usually did, because it did not change the outcome, and they had an obligation to provide care to the person anyway and to protect autonomy by avoiding the use of coercion. In this study the understanding of autonomy seemed to be synonymous with self-determination. One aim of modern legislation and guidelines for dementia care is shared decision-making, but previous studies have illuminated that the aim of shared decision-making in care for persons with dementia is often a dilemma and may be difficult to achieve [46, 47]. In persons with dementia, their choices are not always well informed due to cognitive decline [48]. This makes it even more important to assess whether the person in need of care has the possibility to understand their own needs or not, and to understand the impact their choices may have on their health and possible suffering.

The nurses in our study did state that many of their patients could not make well-informed choices and that they did not understand what was good for them. They described situations where they questioned if the person with dementia received needed care. For example, one nurse described a situation where the person had not showered for over a year and had poor self-care. The findings indicate that the nurses often had an opinion about the person’s capacity to consent, but without making it explicit or documenting it, and often without acting on it. A possible consequence of the lack of documentation or awareness of negative capacity to consent is that persons with dementia receive reduced or no care because their resistance is understood in the same way as of the patients who are cognitively capable of making informed decisions and to say no. Cognitively capable patients also have the advantage of being able to change their minds and contact health care services if suffering becomes worse.

Different understandings of what is included in the definition of forced treatment and care may rely on the understandings of the severity of the situation and what type of support alternatives might be available [8]. The law defines coercion as *interventions of treatment and care carried out, despite the resistance of the patient, and/or against the patient’s will or knowledge* [26].. In the present study, for interventions where it would be wrong or too risky not to intervene, the nurses did not necessarily understand the interventions they applied to be coercive. They described these situations as gray areas coercive care. Examples of such situations could be that the person with dementia was pressured to go to the nursing home, to be assisted with personal hygiene, or to be moved from the chair to the bed at night by force because it would be wrong not to. However, such interventions are included in the definition of coercion in the law and should be reported to the health authorities [32]. In the present study, the definition of forced treatment and care seemed to be first and foremost associated with much stronger interventions, like moving someone by force into an ambulance to be placed in a nursing home.

The findings in this study support one main intention of the law namely to reduce coercion and might be taken as an indication that the value of preventing and reducing coercion in legal guidelines has been successfully translated into clinical home health care practice. The findings of this study are thus in line with previous research where patient autonomy is emphasized and the value of respecting choice is strong in home health care to persons with dementia [13, 49].

These findings have to be seen in light of the person-centered care movement in primary care over the last decades, and the shift from paternalistic approaches to care practices where individualization, user participation, and voluntariness in care for persons with dementia has been emphasized in the vocabulary of care guidelines and modern legal regulations for persons with dementia [49–51]. Nurses’ lack of attention to avoiding severe health damage compared to providing trust-building interventions in home health care may be ascribed to less emphasis on the professional responsibility to provide necessary health care in educational dementia care campaigns and courses.

An element to consider in light of the findings regarding the strong dissociation from forced treatment and care is whether the strong moral incentives to avoid coercion in the nursing culture have made it problematic to discuss difficult situations of resistance to care at all. If this is the case, it is a problem because if situations of resistance and forced treatment and care are not acknowledged or made explicit, they will remain morally un-acceptable, un-reportable, and non-transparent. Situations with strong resistance, i.e., to needed pain medication or to needed personal hygiene due to infection, may become stalled and stay unreported because it becomes difficult to speak of them.

The findings demonstrated great challenges in supporting persons with dementia who resist care in their homes. To enable persons with dementia to independently live at home and avoid unwanted placement in a nursing home, balancing health-risks at home needs to get greater attention [34, 42]. Communication about these issues is delicate and challenging, and communication may be utterly constrained if the organizational culture does not acknowledge a need to balance health-risk assessment and safety measures against self-determination and autonomy in persons with dementia.

What the findings of our study did not successfully demonstrate is whether it is plausible to provide needed health care to avoid severe health damage for home-dwelling persons with dementia that strongly resist it. There seems to be an indication that nurses do not always find this possible. However, a care approach where trust-building becomes both the only aim and means of nursing care may imply great suffering due to severe health damage in home-dwelling persons with dementia.

### Limitations

Although a brief introduction about the concept of resistance and the topic was given prior to each interview, the nurses’ subjective perceptions of what should be defined as resistance and forced treatment and care may have influenced the study. Forced treatment and care was intentionally not defined in the introduction, because we wanted the nurses to describe their experiences of what happened when the encounter resistance to care

## Conclusion

Our findings indicate that nurses’ responsibility to decide how to conduct care is downplayed when facing resistance to care. Further, their professional judgement when encountering resistance to care from home-dwelling persons with dementia is influenced by contextual factors and characterized by a strong commitment to avoid forced treatment and care, usually by reducing and adapting care to the circumstances. To secure professional services in future home health care it is crucial the nurses’ roles and responsibilities are clearly communicated.

A continuous challenge for future research and dementia care is how nursing decisions when faced with resistance can safeguard shared decision-making at the same time as they are balanced against risks of severe health damage in home-dwelling persons with dementia. A fundamental question to ask is whether autonomy does conquer all, even when severe health damage is at stake.

## Data Availability

The datasets analyzed during the current study are not publicly available due to lack of permission to publish raw data from the participants. The nature of the data collected implies that anonymity would be difficult to secure. The data are therefore not made public for ethical reasons. Anonymized quotes in the manuscript aids safeguard unethical or incorrect conclusions drawn from the data.
